# Plasma metabolomics profiling of 580 patients from an Early Detection Research Network prostate cancer cohort

**DOI:** 10.1038/s41597-023-02750-7

**Published:** 2023-11-25

**Authors:** Elisa Benedetti, Kelsey Chetnik, Thomas Flynn, Christopher E. Barbieri, Douglas S. Scherr, Massimo Loda, Jan Krumsiek

**Affiliations:** 1 Department of Physiology and Biophysics, Weill Cornell Medicine, New York, NY USA; 2 Englander Institute for Precision Medicine, Weill Cornell Medicine, New York, NY USA; 3 Department of Urology, Weill Cornell Medical College, New York-Presbyterian Hospital, New York, NY USA; 4 Department of Pathology and Laboratory Medicine, Weill Cornell Medicine, New York, NY USA; 5 Meyer Cancer Center, Weill Cornell Medicine, New York, NY USA

**Keywords:** Prostate cancer, Cancer

## Abstract

Prostate cancer is the second most common cancer in men and affects 1 in 9 men in the United States. Early screening for prostate cancer often involves monitoring levels of prostate-specific antigen (PSA) and performing digital rectal exams. However, a prostate biopsy is always required for definitive cancer diagnosis. The Early Detection Research Network (EDRN) is a consortium within the National Cancer Institute aimed at improving screening approaches and early detection of cancers. As part of this effort, the Weill Cornell EDRN Prostate Cancer has collected and biobanked specimens from men undergoing a prostate biopsy between 2008 and 2017. In this report, we describe blood metabolomics measurements for a subset of this population. The dataset includes detailed clinical and prospective records for 580 patients who underwent prostate biopsy, 287 of which were subsequentially diagnosed with prostate cancer, combined with profiling of 1,482 metabolites from plasma samples collected at the time of biopsy. We expect this dataset to provide a valuable resource for scientists investigating prostate cancer metabolism.

## Background & Summary

Prostate Cancer (PCa) is the second most common malignancy (after lung cancer) in men, with more than 1.4 million new cases and more than 375,000 deaths worldwide in 2020^1^. When detected early, PCa can usually be treated and managed without impacting the patient’s lifespan, and the 5-year survival rate for patients diagnosed with early-stage, localized PCa is virtually 100%^2^. However, when detected after the appearance of symptoms, PCa is typically at a more advanced stage, often after the development of metastases. For this group of patients, the 5-year survival rate drops to 32%^3^. Therefore, early detection of this disease is essential for patient survival.

Serum Prostate-Specific Antigen (PSA) quantification has been at the core of screening practices for the past 35 years^4^. However, recent studies have shown how PSA, while being informative in the prediction of recurrence^5,6^, has demonstrated very limited accuracy in discriminating PCa patients from controls (only ~25% of men with elevated PSA are found to have prostate cancer after biopsy^7^), and in discriminating indolent from aggressive disease (Area-under-the-curve, or AUC, less than 65%^8^). This has led to substantial overdiagnosis and overtreatment of patients with indolent disease^9,10^, with significant impact on the quality of life of these patients^11^. Therefore, better biomarkers for the early detection of PCa are urgently needed.

The National Cancer Institute Early Detection Research Network (NCI-EDRN) Prostate Cancer Cohort has been created to collect patient specimens and develop new clinically applicable molecular biomarkers to replace PSA testing for the diagnosis and risk assessment of PCa^12^. As part of these efforts, Weill Cornell Medicine recruited 1,144 patients with no prior history of prostate cancer, who underwent a prostate biopsy between 2008 and 2017. Extensive clinal data were collected for these patients, including patient’s demographic information and medical history, as well as 12- and 24-month clinical follow-up. For cancer patients, this included details on patient treatment and response, additional longitudinal PSA measurements, as well as the results of any relevant clinical tests, such as CTs or bone scans, MRIs, or repeat biopsies. For controls, information regarding longitudinal PSA measurements, digital rectal exam (DRE) results, and repeat biopsy results were recorded where available.

Recent studies have identified several blood metabolites as potential biomarkers for PCa diagnosis and prognosis. Among these, elevated sarcosine levels and decreased levels of phosphatidylcholines (PCs) and citrate have been observed in plasma of PCa patients compared to healthy controls^13,14^. Moreover, increased sarcosine abundance has been linked to prostate cancer metastasis^15^. A notable study employed 1H NMR-based metabolomics to discern characteristic metabolic panels for different stages of prostate cancer, demonstrating the potential of metabolomics in cancer research^16^. The discovery of these biomarkers is crucial for more precise PCa diagnosis^13,17^. However, further validation is needed to ensure their clinical effectiveness^18^.

**Fig. 1:**
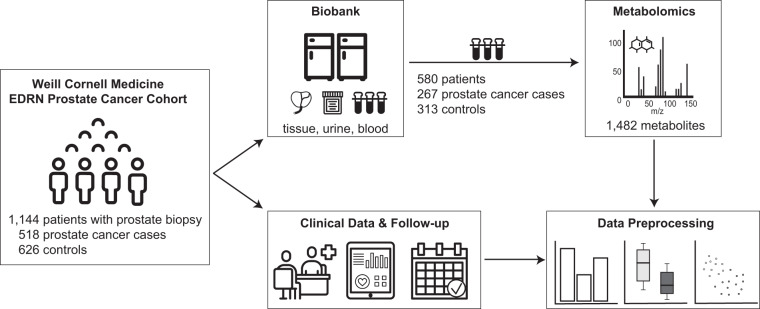
Study overview. The Weill Cornell Medicine EDRN Prostate Cancer Cohort included 1,144 patients undergoing prostate biopsy. For each patient, biopsy tissue, as well as urine and blood plasma were collected and biobanked. Clinical annotations included demographic information and medical history, as well as 12- and 24-months follow-up including biopsy pathology reports, PSA measurements, patients’ treatment, and clinical exams. From this cohort, plasma samples of 580 patients (267 cases and 313 controls) were profiled metabolically by Metabolon, Inc.

Here, we present a new dataset related to 580 individuals from the Weill Cornell EDRN Prostate Cancer Cohort, for which metabolomic profiling was performed on plasma samples collected prior to biopsy (Fig. 1). The metabolite quantification included four profiling modes: (1–2) two separate reverse phase (RP)/Ultrahigh Performance Liquid Chromatography (UPLC) – Tandem Mass Spectroscopy (MS/MS) methods with positive ion mode electrospray ionization (ESI), (3) one RP/UPLC-MS/MS with negative ion mode ESI, and (4) one Hydrophilic Interaction Chromatography (HILIC) UPLC-MS/MS with negative ion mode ESI, allowing for the detection of more than 1,400 compounds, spanning 9 molecular classes and almost 120 molecular pathways. Here we make these data, together with the corresponding rich clinical annotations, publicly available to the scientific community.

## Methods

### Weill cornell early detection research network (edrn) prostate cancer cohort

The Weill Cornell EDRN Prostate Cancer Cohort was consented and recruited 1,144 patients (518 cases and 626 controls) over 9 years (from 2008 to 2017). In order to be eligible for the study, patients needed to be adult males, have no prior history of prostate cancer or prostate biopsies, and be scheduled to undergo prostate biopsy at Weill Cornell Medicine in New York City. Need of prostate biopsy was determined based on suspicion of prostate cancer, which primarily included elevated PSA levels, abnormal digital rectal exam (DRE) results, or suspicious findings based on imaging. Prostate biopsies included at least 10 cores taken in a laterally directed fashion, and a fasting blood sample was collected prior to prostate biopsy from all recruited patients. Biopsy tissue, together with urine and blood samples were processed and biobanked at Weill Cornell Medicine.

For each patient, extensive clinical parameters were collected, including biopsy results, prostate cancer diagnosis and 2-year follow-up clinical information. EDTA-plasma samples for metabolomics profiling were selected to include patients with complete 12- and 24-months follow-up information available (see Data Records section for more details). This subset included a total of 580 patients, with 267 men diagnosed with PCa and 313 controls (Fig. 1). An overview of the main clinical and demographic parameters for this cohort is provided in Table 1.

**Table 1:** Demographics of the profiled cohort.

	Cases (N = 267)	Controls (N = 313)	p-value
Age in years			0.109
median (IQR)	66.0 (59.0–71.5)	64.0 (58.0–70.0)	
[min, max]	[39, 85]	[33, 84]	
BMI			0.981
median (IQR)	26.3 (24.4–29.0)	26.3 (24.2–19.3)	
PSA in ng/ml			0.010
median (IQR)	5.3 (4.0–7.9)	4.9 (3.6–6.7)	
Elevated PSA	N = 242	N = 276	0.490
Abnormal DRE	N = 40	N = 21	0.001
Gleason score (GS)	GS 6 N = 113 GS 7 N = 121 GS 8 N = 15 GS 9 N = 17 GS 10 N = 1	—	—

BMI = Body Mass Index; PSA = Prostate-Specific Antigen; DRE = Digital Rectal Exam; IQR = Interquartile Range. A Wilcoxon test was performed for continuous variables (Age, BMI, PSA), and a Fisher’s exact test for dichotomous variables (Elevated PSA, Abnormal DRE).

This study has been approved by the Weill Cornell Internal Review Board (IRB protocol number 0711009545). All patients provided informed consent for the collection, storage, and de-identified sharing of their data for research purposes. The IRB has approved the release of data in the provided format.

### Sample collection processing

Fasting blood was collected prior to biopsy in 6 ml BD Vacutainer**®** EDTA tubes. Immediately after blood draw, the tube was inverted 8–10 times to mix the additive with the blood and placed immediately on ice. Tubes were centrifuged at 2,500 rpm for 15 minutes at 4 °C within two hours of blood collection. Plasma aliquots of 100ul or 200 ul were extracted from the top layer, transferred into 0.5 ml polypropylene micro tubes and stored at −80C until shipment for metabolomic profiling.

### Metabolomics analysis

Metabolomic profiling of samples and quality control was performed by Metabolon Inc. (Morrisville, NC).

### Sample preparation

Upon receipt, samples were catalogued and instantly stored at −80oC until they were processed. Each acquired sample was allocated a unique identifier for tracking all handling, tasks, and outcomes. Both the original samples and any resultant aliquots were monitored continuously through an automated system.

On the extraction day, the frozen samples were thawed while on ice. Sample preparation was conducted using the automated MicroLab STAR® mechanism (Hamilton Company, Reno, NV). A volume of 100 µl from each sample was relocated to a well within a deepwell plate. Before the extraction, several isotopically labeled standards were introduced to each specimen to ensure precise extraction. For the purpose of removing proteins or disengaging small molecules bound to proteins or encapsulated in the precipitated protein matrix, proteins were sedimented with 500 µl of methanol via rigorous shaking for 2 minutes using a GenoGrinder 2000 (Glen Mills, Inc, Clifton, NJ), succeeded by a 10-minute centrifugation at 680 g.

### Ultrahigh performance liquid chromatography – tandem mass spectroscopy (UPLC-MS/MS)

The sample was partitioned into five aliquots: two for examination using dual independent reverse phase (RP)/Ultrahigh Performance Liquid Chromatography (UPLC) – Tandem Mass Spectroscopy (MS/MS) approaches with positive ion mode electrospray ionization (ESI), one for testing through RP/UPLC-MS/MS with negative ion mode ESI, one for evaluation via Hydrophilic Interaction Chromatography (HILIC) UPLC-MS/MS with negative ion mode ESI, and a single sample was set aside for backup. Samples were momentarily situated on a TurboVap® (Zymark, Inc., Hopkinton, MA) to eliminate the methanol solvent. Extracts were subsequently dried under liquid nitrogen cooling prior to analysis.

Chromatography was performed using an ACQUITY (Waters, Milford, MA) ultra-performance liquid chromatography (UPLC) held at 40 °C–50 °C, and mass spectrometry was performed using a Q-Exactive (Thermo Scientific, Waltham, MA) high resolution/accurate mass spectrometer interfaced with a heated electrospray ionization (HESI-II) source and Orbitrap mass analyzer operated at 35,000 mass resolution. The linearity of the instrument performance standards has been shown previously^19^. Immediately prior to analysis, dried samples were reconstituted in 40 µL ›of the “A” mobile phase solvent, as specified for each analytical method in the supplementary data of Ford *et al*.^20^. Each reconstitution solvent contained several instrument performance standards at fixed concentrations to ensure injection and chromatographic consistency and to aid in peak alignment. After reconstitution, samples were centrifuged at 2800 rpm for 5 minutes. The injection volume was 5 mL with a 2x loop overfill.

For the RP/UPLC - MS/MS with positive ion mode ESI arm, the two aliquots were analyzed using acidic positive ion conditions; one was chromatographically optimized for more hydrophilic compounds and the other for more hydrophobic compounds. In the first case, the extract was gradient-eluted from a C18 column (Waters UPLC BEH C18-2.1 × 100 mm, 1.7 µm) using water and methanol, containing 0.05% perfluoropentanoic acid (PFPA) and 0.1% formic acid (FA), while in the second, the extract was gradient-eluted from the same C18 column mentioned before, this time using methanol, acetonitrile, water, 0.05% PFPA and 0.01% FA and was operated at an overall higher organic content (for details, see Ford *et al*.^20^).

The aliquot for profiling with RP/UPLC-MS/MS with negative ion mode ESI was analyzed using basic negative ion optimized conditions, using a separate dedicated C18 column. The basic extracts were gradient-eluted from the column using methanol and water, with 6.5 mM Ammonium Bicarbonate at pH 8.

The aliquot for profiling with HILIC/UPLC-MS/MS with negative ion mode ESI was analyzed via negative ionization following elution from a HILIC column (Waters UPLC BEH Amide 2.1 × 150 mm, 1.7 µm) using a gradient consisting of water and acetonitrile with 10 mM Ammonium Formate, pH 10.8. The MS analysis alternated between MS and data-dependent multistage (MSn) scans using dynamic exclusion. The scan range varied slighted between methods but covered 70–1000 m/z.

All MS2 spectra were collected with a data dependent acquisition (DDA) method using an exclusion list as described in Ford *et al*. previously^20^.

Detailed instrument parameters, reagents, standards, and chromatography run times are listed in Supplementary Material 1.

### Data extraction and compound identification

Proprietary, in-house software by Metabolon was used to perform the detection and integration of MS peaks. The extracted chromatograms were binned by mass in a specified range; moreover, for each sample, a noise baseline was determined. All samples were aligned using the retention index (RI)^21^, a parameter computed from retention time (RT), of internal standards spiked into every analyzed sample. The internal standards were isotopically labelled metabolites that elute approximately every 30 s of chromatography, therefore spanning the whole chromatographic range, providing a reference for alignment across samples.

Compound identification was performed by matching the ion chromatographic features to a curated library of more than 5,000 purified standards or recurrent unknown entities^22^. Biochemical identifications were based on three criteria: (1) retention index within the RI window of the proposed identification, where the window is based both on the behavior of standards over a concentration range as well as historic behavior, (2) accurate mass match to library entries within 10 ppm m/z of the measured compound, and (3) a forward and reverse fragmentation scores^23^. Briefly, the forward score indicates how well the ions in the experimental spectrum were present in the library at the correct ratios, while the reverse score indicates how well the ions in the library were present in the experimental spectrum at the correct ratios. Compounds that satisfied criteria 1 and 2 and with forward and reverse fragmentation scores above 80% were automatically approved, while compounds with fragmentation scores below 35% were automatically rejected. Compounds with intermediate scores were marked for manual review and approval^22^. Metabolites were assigned confidence tiers according to the following guidelines: Biochemical Name (no asterisk) corresponds to Metabolomics Standards Initiative Tier 1 identification, indicating a compound confirmed based on an authentic chemical standard with high confidence in its identity. Biochemical Name * indicates a compound that has not been confirmed based on a standard, but for which there is high confidence in its identity (Not Tier 1). Biochemical Name ** represents a compound for which a standard is not available, but for which there is reasonable confidence in its identity or the information provided (Not Tier 1).

Peaks that could not be matched to any entry in the library were individually analyzed across injections, to identify strong correlations likely arising from isotope or adduct relationships. Recurrent peaks that could not be related to known compounds but that strongly correlated across multiple injections were considered likely to represent authentic biological entities that are not yet included in the library entries. These compounds were manually analyzed and reviewed, and, if considered to likely represent a novel undocumented biochemical, were assigned a numerical designation (e.g., “X-12345”) and were added as a new library entry for future analyses and classification. Importantly, these unknown metabolite names are consistent across studies, allowing for comparability with other Metabolon datasets.

### Curation

All identification and quantification tasks underwent QC to confirm the quality of the identification and the integrity of peak integration. This concluding verification stage eliminated procedural artifacts, substances with inferior peak forms and thus flawed integration, compounds showing a systematic upward or downward trend in area, and fragmentation scores that led to rejected library identifications. Procedural artifacts were categorized as biochemicals existing in the biological samples at levels < = 3× those in the water process blanks. For compounds that were detected in both positive and negative modes, one mode was selected to represent the substance, based on the relative standard deviation among samples and the percentage of samples where the metabolite was observed in each mode.

### Data normalization

Since the samples in this study were profiled over multiple days (run-days), a data normalization step was performed to correct variation resulting from inter-day instrument tuning differences. The integrated area-under-the-curve data for each metabolite was first divided by the corresponding run-day median for that metabolite and then multiplied by the overall dataset median for that metabolite.

### Missing values

Missing values can result from chromatographical issues, software processing errors, when metabolite levels are below the limit of instrument detection (LOD), or simply when a metabolite is not present in a particular sample. Alternatively, sometimes missing values arise due to technical problems such as a temporary reduction in electrospray performance due to particulate material in the spray nozzle (Payne *et al*. 2009). These values are represented by NAs (not a number) in the data matrix.

### Data preprocessing

To facilitate statistical analysis, we provide a preprocessed version of the metabolomics data in addition to the raw metabolite ion counts. The preprocessing workflow involved the following steps: (1) Metabolites with more than 50% missing values were filtered out. (2) Metabolite abundances were normalized using the probabilistic quotient approach^24^, using only variables with less than 20% missing values to construct the reference sample. (3) Normalized values were log-scaled to improve normality, as metabolite abundances are typically log-normally distributed. (4) The remaining missing values were imputed using a k-Nearest-Neighbors-based algorithm (knn with variable preselection and k = 10)^25^.

### Clinical annotations

This cohort has rich clinical annotations, which range from global demographic details and patients’ medical history to detailed pathology and treatment information, as well as 12- and 24-month follow-up for both cases and controls, for a total of 420 unique parameters. Importantly, the follow-up information includes longitudinal PSA measurements pre and post biopsy, detailed biopsy pathology reports, patients’ treatment, as well as any additional prostate cancer related clinical test, such as CTs or bone scans, MRIs, or repeat biopsies.

An overview of the types of annotations available is provided in Table 2. A detailed description of all clinical parameters is included in the clinical annotation data file (see Data Records).

**Table 2:** Overview of clinical annotations.

Category	Number of parameters	Examples
Demographics	14	Age
		Ethnicity
		Smoking
Cancer history	8	Family history of prostate cancer
		Previous cancer diagnoses
Medical history/Comorbidities	12	Arthritis
		Diabetes
		Inflammatory bowel disease
Pre-biopsy clinical data	43	Digital rectal exam results
		Longitudinal PSA measurements
		Low % free PSA
Prostate-related conditions	10	Prostatitis
		Benign prostatic hyperplasia
		Urethritis
Prostate-related medications	10	5-alpha reductase inhibitors
		Alpha-blockers
		Androgens
Past surgical history	10	TURP
		TUIP
		TUMT
Biopsy procedure details	7	Biopsy date
		Number of cores taken
		Prostate size
Prostate cancer diagnosis	14	Cancer diagnosis
		Gleason score
		TNM stages
Per-biopsy-core pathology report	97	Core position
		Primary Gleason score
		Secondary Gleason score
		Perineural invasion
		Percent of cancer in core
		Core length
Negative biopsy reporting	5	High grade PIN
		Atypia
		Atrophy
12-month follow-up clinical data	60	Bone scan results
		CT scan results
		MRI results
12-month follow-up prostate cancer treatment details	31	Treatment Type and Duration
		Radical prostatectomy results
24-month follow-up clinical data	62	Bone scan results
		CT scan results
		MRI results
24-month follow-up prostate cancer treatment details	48	Treatment type and duration
		Radical prostatectomy results

## Data Records

The integrated metabolite ion counts, the preprocessed metabolomics data, the metabolite annotations, and the deidentified clinical data are available for download on Metabolomics Workbench, 10.21228/M86H7K^26^. The data is available as a zip file (E*DRN_Data.zip*) and contains two data files (*EDRN_MetabolomicsData.xlsx* and *EDRN_ClinicalData.xlsx*) and one text document (*preprocessing_workflow.docx*) detailing the data preprocessing steps.

The unprocessed ion count data (tab “Data” in file EDRN_MetabolomicsData.xlsx) include metabolite abundances of 1,482 biochemicals, of which 1,155 are compounds of known identity (named biochemicals) covering 119 biochemical pathways across 9 molecular classes (tab “MetaboliteAnnotations” in file EDRN_MetabolomicsData.xlsx), and 327 are compounds of unknown structural identity (unnamed biochemicals). The preprocessed data (tab “PreprocessedData” in file EDRN_MetabolomicsData.xlsx) were generated as described in Section 2.4 and only account for metabolites with less than 50% missing values. The data file includes 1,169 metabolites (915 known and 254 unknowns). To protect patients’ privacy, in the clinical annotation file (tab “ClinicalAnnotations” in file *EDRN_ClinicalData.xlsx*) the age of individuals older than 90 years is reported as 90 and all dates have been reported as number of days prior to or since biopsy. A detailed description of each clinical variable is provided in the tab “Legend”.

## Technical Validation

In order to avoid systematic effects in any of the comparison groups, samples were randomized both across profiling days and within each profiling run and plate.

Moreover, several types of control samples were analyzed in parallel with the experimental samples to assess instrument and process variability. First, a pool of well-characterized EDTA human plasma (“MTRX”) served as a technical replicate throughout the data set. These matrix samples were used to estimate process variability by calculating the median relative standard deviation (RSD) for all measured metabolites (i.e., non-instrument standards) present in 100% of the pooled matrix samples. The median RDS for process variability was around 9%.

Additionally, a mixture of QC standards was spiked into every analyzed sample and used to aid chromatographic alignment and to monitor instrument performance. In this case, instrument variability was determined by calculating the median RDS for the QC standards that were added to each sample prior to injection into the mass spectrometers. The median RDS for instrument variability was roughly 6%. Note that the QC standards were carefully chosen not to interfere with the measurement of compounds in the biological samples.

**Fig. 2:**
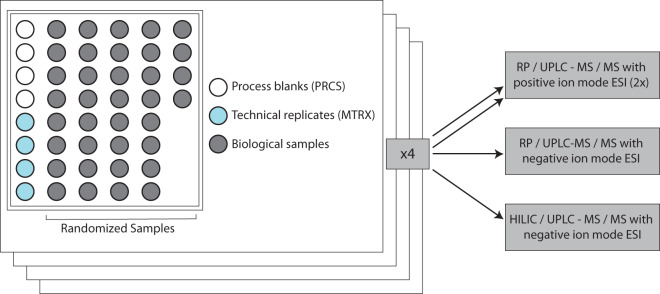
Plate layout and quality control samples. Each plate included four ultra-pure water samples that served as process blanks (PRCS) for the assessment and removal of process artifacts, four samples of pooled EDTA human plasma (MTRX) that served as technical replicates throughout the dataset, and 36 biological samples. For metabolomic analysis, each plate is generated in four replicates, one for each of the four platform arms: two separate reverse phase (RP)/Ultrahigh Performance Liquid Chromatography (UPLC) – Tandem Mass Spectroscopy (MS/MS) methods with positive ion mode electrospray ionization (ESI), one RP/UPLC-MS/MS with negative ion mode ESI, and one Hydrophilic Interaction Chromatography (HILIC) UPLC-MS/MS with negative ion mode ESI.

Water samples (“PRCS”) were additionally included in each run and served as process blanks for the assessment and removal of process artifacts. A schematic representation of the plate layout is provided in Fig. 2.

### Supplementary information

Supplementary Material 1

## Data Availability

The R code used to preprocess the metabolomics data was based on R 4.0.1 and the R package maplet^27^. The code is available at https://github.com/krumsieklab/prostate-cancer-edrn. In the same script, we also provide an example to illustrate how to use the maplet R package to load the data and run differential analyses based on the available clinical parameters. This can serve as a template for users to build their own analysis pipelines.
