# Supplementary material for: Plasma metabolomics profiling of 580 patients from an Early Detection Research Network prostate cancer cohort
Source: Sci Data. 2023 Nov 25;10:830. doi: 10.1038/s41597-023-02750-7 (PMC10676366; doi:10.1038/s41597-023-02750-7)
Supplement: Supplementary file 1 — Supplementary Material 1 [file 41597_2023_2750_MOESM1_ESM.pdf]

Supplementary Material:
Chromatography and Mass Spectrometry Conditions

These measurement conditions have previously been described in: Ford et al., *The Journal of Applied Laboratory Medicine*, Volume 5, Issue 2, March 2020, Pages 342–356,
https://doi.org/10.1093/jalm/jfz026

1 Chromatographic Conditions

1.1 LC/MS/MS Pos Polar

Parameter	Value
Column	Waters BEH C18 1.7um 2.1x100mm
Mobile Phase A	0.1% formic acid and 0.05% PFPA in water, pH ~2.5
Mobile Phase B	0.1% formic acid and 0.05% PFPA in methanol, pH ~2.5
Flow Rate	0.35 mL/min
Gradient Elution	Linear gradient from 5% B to 80% B over 3.35 minutes.

1.2 LC/MS/MS Pos Lipid

Parameter	Value
Column	Waters BEH C18 1.7um 2.1x100mm
Mobile Phase A	0.1% formic acid and 0.05% PFPA in water, pH ~2.5
Mobile Phase B	0.1% formic acid and 0.05% PFPA in 50% methanol/50% acetonitrile, pH ~2.5
Flow Rate	0.60 mL/min
Gradient Elution	Linear gradient from 40% B to 99.5% B over 1.0 minute, hold 99.5% B for 2.4 minutes.

1.3 LC/MS/MS Neg

Parameter	Value
Column	Waters BEH C18 1.7um 2.1x100mm
Mobile Phase A	6.5mM ammonium bicarbonate in water, pH 8
Mobile Phase B	6.5mM ammonium bicarbonate in 95% methanol/5% water
Flow Rate	0.35 mL/min
Gradient Elution	Linear gradient from 0.5 to 70% B over 4.0 minutes, then rapid gradient to 99% B in 0.5 minutes.

1.4 LC/MS/MS Polar

Parameter	Value
Column	Waters BEH Amide 1.7um 2.1x150mm
Mobile Phase A	10mM ammonium formate in 15% water/5% methanol/80% acetonitrile (effective pH 10.16 with NH_4OH)
Mobile Phase B	10mM ammonium formate in 50% water/50% acetonitrile (effective pH 10.60 with NH_4OH)
Flow Rate	0.50 mL/min
Gradient Elution	Linear gradient from 5% B to 50% B in 3.5 minutes, then linear gradient from 50% B to 95% B in 2 minutes.

2 Instrument Performance and Process Assessment Standards

Standards	LC/MS/MS Pos Polar	LC/MS/MS Pos Lipid	LC/MS/MS Neg	LC/MS/MS Polar
Instrument Performance Standards	d7-glucose, d5-glutamine, d2-threonine, d5-hippuric acid, d3-methionine, d3-leucine, Br-phenylalanine	Br-phenylalanine, d5-androstene, d9-progesterone, d4-dioctyphthalate	d7-glucose, d3-methionine, d3-leucine, d8-phenylalanine, d5-tryptophan, Br-phenylalanine, d15-octanoic acid, d19-decanoic acid, d27-tetradecanoic acid, d35-octadecanoic acid, d2-eicosanoic acid	d35-octadecanoic acid, d5-indoleacetate, Br-phenylalanine, d5-tryptophan, d4-tyrosine, d3-serine, d3-aspartic acid, d7-ornithine, d4-lysine
Process Assessment Standards	fluorophenylglycine, chlorophenylalanine	d6-cholesterol, chlorophenylalanine	tridecanoic acid, chlorophenylalanine	fluorophenylglycine, chlorophenylalanine

3 Mass Spectrometry Conditions

Parameter	LC/MS/MS Neg	LC/MS/MS Pos Polar	LC/MS/MS Pos Lipid	LC/MS/MS Polar
Spray Voltage (V)	3300	4000	4200	3000
Mass range (m/z)	80-1000	70-1000	110-1000	80-1000
Sheath Gas (au)	70	70	35	60
Auxiliary Gas (au)	15	35	35	20
Source Temp (°C)	300	300	400	300
Ion Transfer Tube Temp. (°C)	250	250	320	250
Norm. Collision Energy (au)	52,65,78	52,65,78	52,65,78	48,60,72
MSA GC target (au)	1e6	1e6	1e6	1e6
MS Max Fill Time (ms)	60	60	60	60
MSn Ion Target (au)	2e5	2e5	2e5	2e5
MSn Max Fill Time (ms)	120	120	120	120
MSn Isolation Window (m/z)	3	3	3	3
MSn Dynamic Exclusion Time (s)	3	3	3	3
S-Lens RF Level	40	40	50	25
